# Comparison of oral versus intra-articular tranexamic acid in enhanced-recovery primary total knee arthroplasty without tourniquet application: a randomized controlled trial

**DOI:** 10.1186/s12891-018-1996-8

**Published:** 2018-03-15

**Authors:** Duan Wang, Hui Zhu, Wei-Kun Meng, Hao-Yang Wang, Ze-Yu Luo, Fu-Xing Pei, Qi Li, Zong-Ke Zhou

**Affiliations:** 1 Department of Orthopedics, West China Hospital/West China School of Medicine, Sichuan University, 37# Wuhou Guoxue road, Chengdu, 610041 People’s Republic of China; 2 Out-patient department, West China Second University Hospital, Sichuan University, Chengdu, 610041 People’s Republic of China; 3 Key Laboratory of Birth Defects and Related Disease of Woman and Children (Ministry of Education), West China Second University Hospital, Sichuan University, Chengdu, 610041 People’s Republic of China

**Keywords:** Total knee arthroplasty, Tranexamic acid, Oral, Intra-articular, Blood loss

## Abstract

**Background:**

Although randomized controlled trials have confirmed oral tranexamic acid (TXA) can provide similar blood-sparing efficacy compared with intravenous (IV) TXA in total knee arthroplasty (TKA), some concerns do remain about thromboembolic events after such systemic administration. Many studies have confirmed that intra-articular (IA) application of TXA can show similar blood-saving efficacy with minimal levels of systemic absorption compared with IV TXA. However, it remains unclear whether the efficacy and safety of oral TXA administration is equal to or less than that of IA administration in TKA without the use of a tourniquet and drain. Thus, this study was to verify non-inferior efficacy and safety of oral TXA compared with IA TXA in primary TKA.

**Methods:**

A double-blind, randomized, controlled trial was performed to compare three oral doses of TXA (2 g of TXA 2 h before incision, and 1 g of TXA 6 and 12 h after surgery, respectively) with IA TXA (3 g of TXA in 100 mL of saline solution). One hundred forty-seven patients scheduled for TKA were randomized to one of the two interventions. The primary outcome was total blood loss. The secondary outcomes included reduction of hemoglobin concentration, clinical outcomes, blood coagulation values, thromboembolic complications, and transfusion rates.

**Results:**

The mean total blood loss was 788.8 mL in the oral TXA group compared with 872.4 mL in the IA TXA group, with no statistical significance (*p* > 0.05). There were no significant differences in reduction of hemoglobin level, blood coagulation level, and clinical outcomes. The transfusion rates were 4% in oral group and 5% IA group, respectively. Also, no significant differences were identified in thromboembolic complications.

**Conclusion:**

Oral TXA according to the described protocol demonstrated non-inferiority for primary TKA, with no safety concerns and a greatly reduced cost, compared with the IA TXA. This randomized controlled trial supports the oral administration of TXA in TKA.

**Trial registration:**

The trial was registered in the Chinese Clinical Trial Registry (ChiCTR-INR-17010968) dated 23rd March 2017.

## Background

Total knee arthroplasty (TKA) is viewed as one of the most successful orthopedic surgeries that relieves pain and improves function but is associated with excessive perioperative blood loss that might lead to anemia and blood transfusions [1–4]. Allogeneic transfusion may result in several undesirable adverse events, but not limited to, including infection, heart failure, immunologic reaction, and myocardial infarction, and as a consequence involve increased morbidity and mortality and additional health care costs [5–7]. Based on a multimodal fast-track methodology at many institutions, numerous blood-saving strategies have been suggested to successfully reduce surgery-related blood loss and minimize the risk of post-operative transfusions, such as perioperative blood salvage, autologous blood transfusion, intraoperative hypotensive anesthesia, application of cryotherapy, and use of pharmacologic antifibrinolytics such as tranexamic acid (TXA) [8–11].

Tranexamic acid, an antifibrynolitic medication, can prevent plasminogen activation by blocking the lysine binding site of plasminogen and inhibiting the formation of plasmin, and thereby promote coagulation process [12, 13]. TXA can be administered either intravenously (IV), intra-articularly (IA), or orally in the setting of TKA. Oral administration of TXA had beneficial blood- and cost-saving effect, as confirmed in some randomized controlled trials (RCT), and also reduced transfusions without increased risk of thromboembolic complications compared with placebo [14, 15]. Compared with oral or IV TXA, the IA TXA can provide a maximum concentration of TXA in surgical site and thereby a prolonged effect to reduce postoperative blood loss, but is associated with minimal levels of systemic absorption, which may increase safety. Numerous RCTs [16, 17] and meta-analyses [18] showed valid evidence favoring the effectiveness of various dosages and routes of IA TXA in reducing blood loss and allogeneic transfusion requirements in TKA.

Recent RCTs and meta-analyses have confirmed that oral TXA showed similar blood-sparing efficacy, at a greatly reduced cost, compared with IV TXA [19, 20]. In spite of the potential cost-saving benefits of oral TXA, some concerns do remain about thromboembolic events after systemic administration in high-risk patient population [21]. Many studies have shown the non-inferiority of IV compared with IA TXA regarding both blood loss and thromboembolic complications. However, it is not clear whether the efficacy and safety of oral TXA administration is equal to or less than that of IA administration.

Some studies have demonstrated that tourniquet application may be related to tissue damage, severe thigh pain, and delayed rehabilitation [22]. In view of these concerns, no tourniquet was applied during the perioperative period at our institution. However, little is known about the efficacy and safety of oral TXA when tourniquet is not utilized in the knee surgery due to the substantial effect of tourniquet-related fibrinolysis on bleeding kinetics.

Thus, the objective of our prospective, randomized, double-blind trial, conducted in an enhanced recovery setup at our institution, was to assess the efficacy and safety of oral administration of three doses compared with IA administration of 3 g of TXA in the setting of primary unilateral TKA, with no tourniquet and drain. We hypothesized that oral TXA would be equivalent to IA TXA in reduction of blood loss and transfusion rates without increased thromboembolic complications.

## Methods

This prospective, randomized, controlled trial was conducted at the Department of Joint Surgery in West China Hospital, Sichuan University. Approval was obtained from the Institutional Review Board (No. 201302008), and written informed consent and research authorizations were obtained from all participants. The trial was registered in the Chinese Clinical Trial Registry.

From March 2017 to July 2017, all patients (aged 18 year. or older) with primary osteoarthritis scheduled for undergoing primary unilateral TKA were screened for enrollment. All perioperative managements of TKA were conducted based on a well-established multimodal enhanced-recovery strategy, including pain control [23–25], blood-saving management [26], and early ambulation [27, 28]. The exclusion criteria were secondary osteoarthritis (i.e., rheumatoid arthritis, post-septic arthritis, or post-traumatic arthritis), known allergy to TXA, a history of arterial or venous thromboembolic disease (i.e. deep venous thrombosis (DVT), or pulmonary embolism (PE)), a history of major comorbidities (i.e. severe pulmonary disease, severe renal insufficiency, hepatic failure, or severe stroke), a history of hematopoietic or hemophilia disease or active cancer, participation in another clinical trial during the last year, pregnancy, and alcohol abuse. These patients were also excluded if they declined to participate or refused to receive blood products.

### Drug delivery and randomization

Recruited patients were randomly allocated to two interventions (i.e., oral-only or IA-only) based on a computer-generated randomization list, which was generated with use of Randomization.com.

#### Group 1 (Oral TXA)

Patients assigned to the oral group were given 2 g of TXA (4 tablets of 500 mg) by oral bolus appropriately 2 h before incision as a preoperative dose. A postoperative dose of 1 g was repeated 6 and 12 h after surgery, respectively. Also, the oral group received 100 mL of an intra-articular placebo solution (0.9% physiological saline solution) in a manner identical to the application of the solution in the IA group. Pharmacokinetic studies have demonstrated that 2 g oral TXA reaches therapeutic concentration after approximately 2 to 3 h and remains above the effective levels required to inhibit fibrinolysis for 6 h after such administration [29, 30].

#### Group 2 (IA TXA)

Patients assigned to the IA group received an intra-articular administration of 100 mL of saline solution containing a 3-g dose of TXA on the basis of previous study of topical TXA in TKA showing high efficacy for reducing bleeding with this dosage and concentration [31, 32]. IA TXA (topical study medication) was administered at two points: (1) after all components were cemented and the joint was thoroughly irrigated, half of the volume (50 mL of 1.5-g TXA solution) was applied to soak the open joint surface and tissue for 5 min; (2) the remaining half was administered using a needle to achieve tissue impregnation before capsule closure [32–34]. Moreover, the IA group was given small placebo pills identical to oral TXA in appearance and quantity with no active ingredient 2 h before incision, 6 and 12 h after surgery, respectively.

One experienced surgeon responsible for all TKAs enrolled all participants, and a research personnel prepared patient assignments, recorded practical details, and rechecked inclusion criteria. The randomization assignments were placed into sequentially numbered opaque sealed envelopes, which were kept by a certificated research pharmacist and were inaccessible through the investigation period. An envelope was opened on the day of surgery, and the appropriate study drug and placebo preparations were handled by a research pharmacist not involved in patient care to ensure identical appearance. The patients, trial participants, anesthesiologists, health-care providers, outcome assessors, and data collectors were blinded to allocation and route of TXA administration.

### Surgical procedure and postoperative management

At our institution, the TKA was conducted by the same senior orthopaedic surgeons (XXX) under general anesthesia with a standard medial para-patellar approach. All prostheses were fixed with cement, and patella resurface technique was used in all patients.

All patients received multimodal analgesia consisting of adductor canal block (20 ml 5 g/L ropivacaine and 0.1 mg adrenaline) and periarticular multi-site infiltration (70 ml 2.5 g/L ropivacaine and 0.1 mg adrenaline) [35]. All patients also received a standard analgesia peri-operatively [23, 24]. Antibiotic prophylaxis in all patients was administered intravenously with 1.5 g of cefuroxime half an hour before surgery. No tourniquet, intra-articular drainage tube, and pressure dressing were applied in all patients in our center [22, 36].

### Thromboembolism prophylaxis protocol

Patients received standard venous thromboembolism prophylaxis based on individualized protocol at our institution, including mechanical and chemical thromboprophylaxis. Patients were given mechanical prophylaxis by means of an intermittent inflatable lower-extremity pump on the first day after surgery, and lower-extremity strength training and passive and active physiotherapy were performed under the supervision of a professional physiotherapist. As for chemical prophylaxis, patients received low-molecular-weight heparin (LMWH; Clexane, Sanofi-Aventis, France, 2000 IU) administered subcutaneously appropriately 8 h after surgery and followed by 4000 IU once a day during hospitalization. Rivaroxaban (10 mg, Xarelto, Bayer, Germany) was administered orally once a day for 10 days after discharge if no bleeding events occurred.

### Blood transfusion protocol

Participants were also received the standard practice of blood-transfusion protocol at our institution, which was consistent with the perioperative transfusion guidelines of Chinese Ministry of Health. Blood products were transfused if the hemoglobin (Hb) level < 7 g/dL in patients who were asymptomatic or if Hb level between 7 and 10 g/dL in patients who developed concomitant clinical symptoms (anemia or myocardial ischemia) or if a patient with any anemia-related organ dysfunction regardless of Hb level.

### Outcome assessment

The primary outcome was total blood loss. The estimated blood loss was calculated applying the Gross formula [37]:

$$ \mathrm{Total}\ \mathrm{blood}\ \mathrm{loss}=\mathrm{PBV}\times \left({\mathrm{Hct}}_{\mathrm{pre}}\hbox{-} {\mathrm{Hct}}_{\mathrm{post}}\right)/{\mathrm{Hct}}_{\mathrm{ave}} $$

$$ \mathrm{PBV}={\mathrm{patient}}^{'}\mathrm{s}\ \mathrm{blood}\ \mathrm{volume} $$

$$ {\mathrm{Hct}}_{\mathrm{pre}}=\mathrm{the}\ \mathrm{initial}\ \mathrm{preoperative}\ \mathrm{hematocrit}\ \mathrm{level} $$

$$ {\mathrm{Hct}}_{\mathrm{post}}=\mathrm{the}\ \mathrm{hematocrit}\ \mathrm{level}\ \mathrm{on}\ \mathrm{the}\ \mathrm{morning}\ \mathrm{of}\ \mathrm{the}\ \mathrm{third}\ \mathrm{postoperative}\ \mathrm{day} $$

$$ {\mathrm{Hct}}_{\mathrm{ave}}=\mathrm{the}\ \mathrm{average}\ \mathrm{of}\ \mathrm{the}\ {\mathrm{Hct}}_{\mathrm{pre}}\mathrm{and}\ {\mathrm{Hct}}_{\mathrm{post}} $$

The PBV was assessed according to the formula of Nadler et al. [38]: PBV (mL) = k_1_ x height (m) + k_2_ x weight (kg) + k_3_; k_1_ = 0:3669, k_2_ = 0:03219, and k_3_ = 0:6041 for men; k_1_ = 0:3561, k_2_ = 0:03308, and k_3_ = 0:1833 for women. If a reinfusion or an allogenic transfusion was performed, the volume transfused should be added when calculating total blood loss.

The secondary outcomes included Hb, reduction of Hb concentration, platelet concentration, hematocrit level, coagulation indicators (i.e., prothrombin time, INR, and activated partial thromboplastin time) on postoperative day (POD) 3, intraoperative blood loss, and amount of IV fluid. Other secondary outcomes included postoperative knee function (i.e., the range of motion and knee society score), pain score, quality of recovery (QoR-40), thromboembolic complications occurring ≤90 days after surgery, surgical site infection, transfusion rates, and number of blood units transfused.

The active range of motion of the knee was measured with use of a standard clinical goniometer in a supine position before surgery and on postoperative 1 and 3 months. By the measurement of suction drains contents and surgical swabs, intraoperative blood loss was evaluated [39]. All patients were examined daily for clinical symptoms of DVT during hospitalization, including pain, swelling, tenderness, superficial venous engorgement, and Homan’s sign in the thigh or calf. When a patient has any suspicious symptom of DVT, a diagnostic Doppler ultrasound was applied to exam both lower limbs by senior ultrasound physicians. All adverse events or thromboembolic events were noted during the first 3 months after surgery. All patients stayed in the hospital for a minimum of 3 days.

### Statistical analysis and sample size

Statistical analyses were conducted using SPSS version 21.0 (SPSS Inc., Chicago, IL) software. A two-sided *P* value of less than 0.05 was generally considered statistically significant for all comparisons. Distributions of demographic data, preoperative laboratory values, surgical data, knee function, and primary and secondary outcomes were assessed with summary statistics, including measures of central tendency (means and standard deviations) for quantitative data and numbers and percentages for qualitative data. Independent t-test was used to compare the normal distributed continuous variables, and Wilcoxon Mann-Whitney U test was applied to analyze non-normal distribution or unequal variables. Pearson chi-square test or Fisher exact test was used for categorical variables. Before breaking the randomization code, statistical analyses were conducted blinded.

The sample-size estimate was determined based on the primary outcome (i.e., total blood loss). Sample size calculations were based on the preliminary data with a minimally clinically important difference of 10% and standard deviation of 15%. Based on the abovementioned information, sample size estimation at an alpha (two-tailed) of 0.05, power of 90%, and standard effect size of 0.65 indicated that 62 patients were required for each arm. To compensate for the expected dropouts (20%), 75 patients per group were planned to include in this study.

## Results

### Patients

**Fig. 1:**
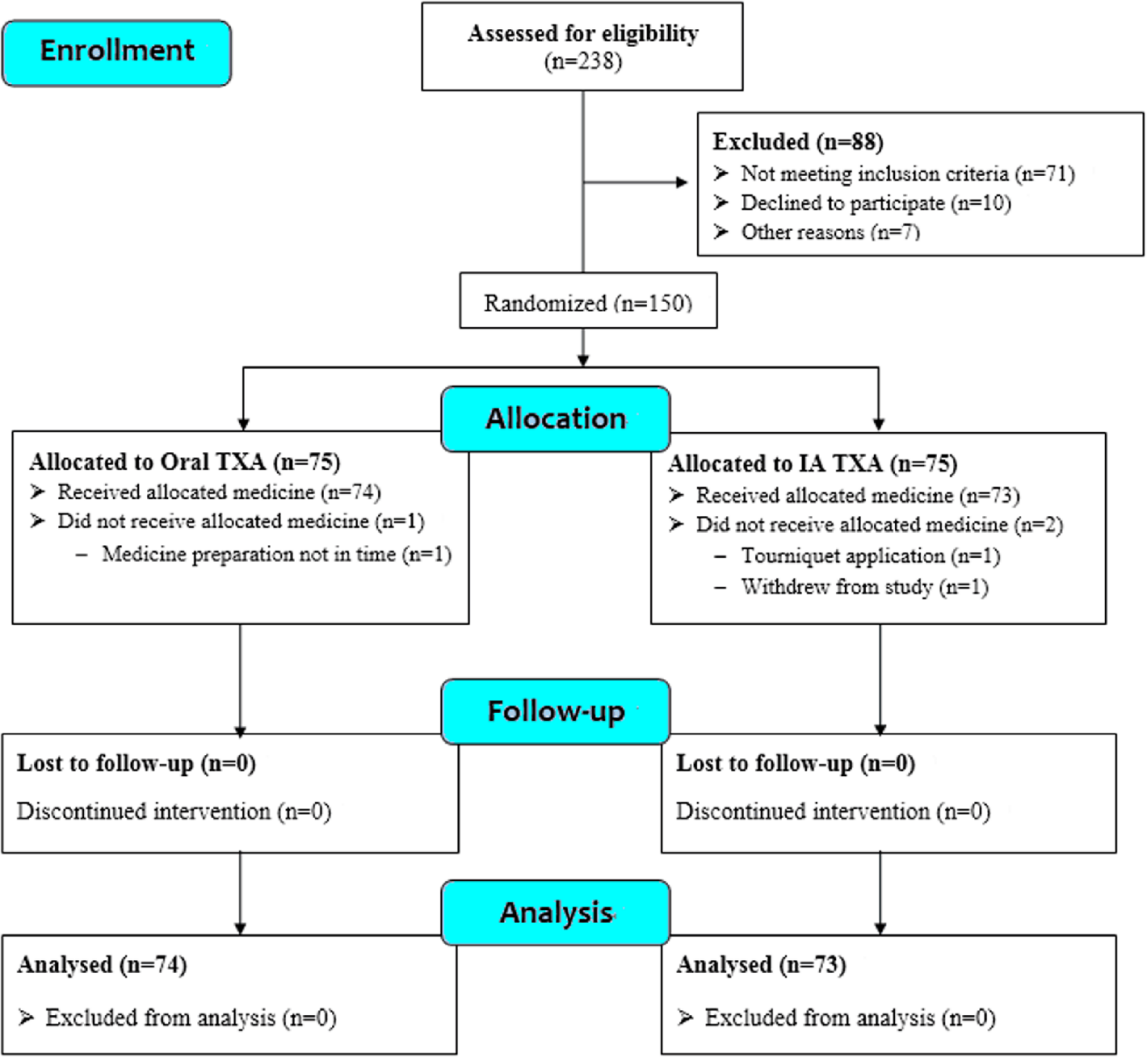
CONSORT (Consolidated Standards of Reporting Trials) flow diagram

During the recruitment period from March 2017 to July 2017, 238 patients scheduled for primary unilateral total knee arthroplasty were screened for participation in this trial. Eighty-eight patients were excluded for the following reasons: 71 were ineligible based on our exclusion criteria, 10 declined to participate, and 7 were excluded due to other reasons (Fig. 1). The remaining 150 patients underwent randomization to receive study medication. Three patients were excluded for various reasons, including tourniquet application (one patient), medication preparation not in time (one), and withdrew consent (one) (Fig. 1). Thus, a total of 147 eligible participants were randomized to receive oral TXA (*n* = 74) or IA TXA (*n* = 73) (Fig. 1). No patient was lost or excluded during follow-up.

No significant differences between the allocation groups were identified with respect to demographic data, perioperative surgical characteristics (i.e., operative time), preoperative Caprini score (i.e., evaluation of individual patients regarding DVT risk based on co-morbidities and risk factors), knee function, and preoperative laboratory values (Table 1). The follow-up duration was 3 months after surgery.

**Table 1:** Baseline characteristics and intraoperative demographics

Variable	Oral TXA Group (*N* = 74)	IA TXA Group (*N* = 73)	*P* value
Patient characteristics			
Age (yr)^b^	65.0 ± 13.1	63.6 ± 11.5	0.51
Gender (Female/Male)^a^	58/16	56/17	0.81
Height (m)^b^	1.5 ± 0.1	1.6 ± 0.2	0.39
Weight (kg)^b^	62.7 ± 11.1	62.9 ± 9.5	0.86
BMI (kg/m^2^)^b^	25.1 ± 4.1	25.5 ± 3.7	0.52
Operated side (L/R)^a^	47/27	43/30	0.57
ASA classification^a^			
I	15 (20%)	12 (16%)	0.79
II	49 (66%)	52 (71%)	
III	10 (14%))	9 (10%)	
IV	0 (0%)	0 (0%)	
Caprini score^b^	8.2 ± 0.9	8.4 ± 1.1	0.27
Preop. laboratory values^b^			
Hemoglobin (g/dL)	13.4 ± 1.3	13.3 ± 1.2	0.40
Hematocrit (L/L)	0.41 ± 0.04	0.41 ± 0.03	0.24
Platelet count (×10^9^/L)	186.4 ± 53.6	189.1 ± 59.1	0.77
Red blood cell count (× 10^12^/L)	4.5 ± 0.5	4.5 ± 0.4	0.63
Prothrombin time (s)	11.6 ± 0.8	11.7 ± 0.9	0.39
INR	0.9 ± 0.1	1.0 ± 0.2	0.28
APTT (s)	27.2 ± 3.4	28.2 ± 3.6	0.10
Fibrinogen (g/L)	2.9 ± 0.9	2.7 ± 0.8	0.24
D-Dimer (mg/L)	0.93 ± 1.1	1.0 ± 1.3	0.68
FDP (mg/L)	2.7 ± 2.1	2.9 ± 3.4	0.62
Surgical data			
Operative time (min)^b^	66.3 ± 10.9	68.8 ± 12.8	0.20
Preop. knee function^b^			
QoR-40	150.9 ± 4.5	151.1 ± 5.0	0.89
ROM	91.8 ± 16.9	92.1 ± 16.7	0.91
KSS	45.4 ± 10.3	47.1 ± 9.5	0.29
Pain VAS score	6.1 ± 1.8	6.2 ± 1.9	0.78
Knee circumference (cm)^b^			
Upper pole of patella	39.9 ± 3.7	39.7 ± 5.5	0.89
Lower pole of patella	33.2 ± 2.8	32.7 ± 3.1	0.25

*ASA* American Society of Anesthesiologists, *PBV* patient’s blood volume, *INR* international normalized ratio, *APTT* activated partial thromboplastin time, *FDP* fibrinogen degradation product, *ROM* range of motion, *KSS* knee society score, *BMI* body mass index, *VAS* visual analogue scale, *QoR-40* quality of recovery-40

^a^Data are presented as number of patients with percentage

^b^Data are presented as Mean ± standard deviation

### Blood loss

The calculated blood loss (primary outcome) was 788.8 ± 349.1 mL in the oral group and 872.4 ± 393.1 mL in the IA group (*p* = 0.21), respectively, with no significant difference between the groups. Regarding secondary outcomes, there were no differences among the groups in terms of intraoperative blood loss (*p* = 0.58) and the amount of postoperative IV fluids on POD 1 (*p* = 0.18) (Table 2).

**Table 2:** Perioperative outcomes regarding blood loss

Variable	Oral TXA Group	IA TXA Group	*P* value
Primary outcome			
Total blood loss (mL)^b^	788.8 ± 349.1	872.4 ± 393.1	0.21
Secondary outcomes			
Intro-operative blood loss (mL)^b^	143.1 ± 25.4	145.6 ± 28.7	0.58
Postop. IV fluid amount (mL)^b^	2729.9 ± 366.5	2818.1 ± 419.2	0.18
Blood transfusion (U)^a^	7	9	0.73
Transfusion rate (%)^a^	3 (4%)	4 (5%)	0.69
Postop. laboratory values at 72 h^b^			
Hemoglobin (g/dL)	11.2 ± 1.3	10.9 ± 1.2	0.22
Reduction of hemoglobin (g/dL)	2.2 ± 0.9	2.4 ± 1.1	0.66
Hematocrit (L/L)	0.34 ± 0.04	0.32 ± 0.03	0.20
Red blood cell count (·×10^12^/L)	3.6 ± 0.4	3.5 ± 0.4	0.37
Platelet count (×10^9^/L)	165.8 ± 53.2	162.2 ± 57.7	0.70
Prothrombin time (s)	11.7 ± 1.3	12.0 ± 1.3	0.17
INR	0.9 ± 0.1	0.9 ± 0.6	0.76
APTT (s)	30.8 ± 5.0	31.4 ± 3.8	0.38
Fibrinogen (g/L)	4.5 ± 1.0	4.4 ± 1.3	0.67
D-Dimer (mg/L)	3.4 ± 2.2	3.2 ± 1.9	0.60
FDP (mg/L)	9.2 ± 6.4	9.1 ± 8.0	0.96

*TXA* tranexamic acid, *INR* international normalized ratio, *APTT* activated partial thromboplastin time, *FDP* fibrinogen degradation product

^a^Data are presented as number of patients with percentage

^b^Data are presented as Mean ± standard deviation

### Postoperative laboratory values and clinical outcomes

No difference between the two groups was identified regarding Hb level on POD 3 (Table 2). The reduction of Hb concentration on POD 3 were 2.2 ± 0.9 g/dL in oral group and 2.4 ± 1.1 g/dL in IA group, respectively, with no significant difference. Also, there were no significant differences identified regarding platelet count, hematocrit level, and red blood cell count on POD 3. In addition, blood coagulation values were similar between the groups (Table 2).

Moreover, knee range of motion and knee society score were similar among the groups at the three-month follow-up visit. Similarly, postoperative pain declined daily, and no significant difference was identified between groups at any follow-up point. When it comes to the severity of the knee swelling, there was no difference between the two groups at postoperative 3 months (Table 3). Moreover, there was no significant intergroup difference observed in terms of QoR-40 during the three-month follow-up visit (Table 3).

**Table 3:** Postoperative outcomes regarding complications and knee function

Variable	Oral TXA Group	IA TXA Group	*P* value
Postop. Complications (%)^a^	2 (2.7%)	1 (1.3%)	0.76
DVT	1	0	0.51
PE	0	0	–
Superficial infection	0	0	–
Hematoma	0	1	0.50
Wound secretion	1	0	0.51
Gastric hemorrhage	0	0	–
Postop. knee function^b^			
QoR-40^b^			
1 M	181.9 ± 5.4	183.4 ± 6.6	0.11
3 M	189.4 ± 5.9	190.1 ± 3.4	0.34
ROM ^b^			
1 M	113.9 ± 8.7	112.6 ± 8.2	0.37
3 M	122.4 ± 10.4	124.4 ± 9.13	0.17
Pain score^b^			
POD 1	4.2 ± 2.3	4.5 ± 1.5	0.39
POD 3	2.4 ± 1.4	2.6 ± 1.5	0.37
3 M	1.4 ± 1.1	1.3 ± 1.2	0.71
KSS ^b^	84.6 ± 4.8	85.9 ± 7.1	0.18
Knee circumference (cm)^b^			
Upper pole of patella	41.2 ± 3.2	41.8 ± 6.0	0.46
Lower pole of patella	35.5 ± 2.4	36.0 ± 3.2	0.28
All cause 30-day mortality^a^	0	0	–
All cause 90-day readmission^a^	0	0	–

*ROM* range of motion, *QoR-40* quality of life-40, *DVT* deep vein thrombosis, *PE* pulmonary embolism, *KSS* knee society score

^a^Data are presented as number of patients with percentage

^b^Data are presented as Mean ± standard deviation

### Blood transfusion

Seven patients received allogeneic blood transfusion due to a postoperative Hb of < 7 g/dL, including three patients (4%) in the oral group and four (5%) in the IA group, respectively, with no significant intergroup difference (Table 2). Similarly, there were no differences regarding the number of units of packed red blood cells transfused.

### Postoperative complications

The frequency of DVT manifestations did not differ significantly between the groups, with 1 case in the oral group compared with none in the IA group. The diagnose of DVT was confirmed by Doppler ultrasonography. However, this patient showed no DVT-related clinical symptoms and was discharged and managed based on usual thromboembolism prophylaxis protocol at our center (Table 3) [32]. No superficial vein thrombosis and PE occurred in any group.

There was one adverse event in the oral group (one wound secretion) and one in the IA group (one hematoma), respectively. No superficial infection and gastric hemorrhage occurred in either group during follow-up period (Table 3). All adverse events were successfully resolved.

## Discussion

Growing evidence has confirmed the efficacy of TXA in reducing blood loss and transfusion rates without additional complications [40, 41]. Our study was conducted to compare the effects of TXA regarding blood loss, transfusions and thromboembolic events, when administered by oral or IA modalities. Several studies have compared the efficacy of oral and IV use of TXA in TKA, while others have compared the effects of IA and IV administration of TXA. The present study was the first that we know of to evaluate two different routes of TXA administration in an enhanced-recovery setup without use of a tourniquet and postoperative drains in TKA. The major finding of this study was that oral administration of three doses of TXA was not inferior to IA administration of 3-g dose with respect to blood loss.

There are several limitations in our study. First, this trial had no placebo group, because the efficacy of TXA has been confirmed in many studies. In addition, the study patients receiving no TXA would be exposed to risks of no beneficial effects of TXA administration, such as reduction in blood loss and the need for transfusions, which may raise ethical issues. Second, we excluded patients undergoing bilateral or revision knee arthroplasty due to much larger blood loss compared with primary TKA; thus, our findings may not be suitable for these patients. Third, the duration of follow-up (3 months) may be short in this study; however, it was adequate to observe associated adverse reactions in three-month follow-up period, as the biological half-life of an intravenous TXA dose is 1.9 to 2.7 h, 90% of which was excreted within 24 h. In addition, blood coagulation concentrations were also monitored for the risk of thrombogenesis postoperatively, which did not differ between groups. The finding was consistent with the previous study [42]. Fourth, no blood analyses were conducted to estimate serum tranexamic acid levels, and thus no toxicity-related information can be provided after such TXA administration following TKA. Fifth, some confounding issues, such as complexity of surgical techniques and extent of soft tissue release, may have a substantial effect on the postoperative blood loss. However, these effects may be negligible due to the randomization design.

Tranexamic acid, an antifibrinolytic agent, can inhibit fibrinolysis though competitively inhibiting plasminogen activation and blocking the binding of plasminogen to fibrin and thereby prevent bleeding. At our institution, retrospective studies containing thousands of patients undergoing TKA revealed no increase in thromboembolic events rates [43]. TXA can be administered intravenously, intra-articularly, and orally. However, the majority of prior studies focused on IV or IA modalities of administration in TKA. However, concerns about the safety of IV and oral administration of TXA and the risk of thromboembolic events still remain for high-risk patient population, who has a history of a thromboembolic event, acute myocardial infarction, or ischemic cerebrovascular accident. In view of these safety concerns, topical application of TXA may be a safer route of administration to reduce postoperative bleeding without increasing the hypercoagulable state associated with knee surgery. IA use of TXA in surgical site can directly target the site of bleeding, achieve surgical hemostasis, and thereby inhibit local activation of fibrinolysis stimulated by surgical site. Also, high topical TXA dose can lead to greater thrombus formation and lower time to vascular occlusion, and result in enhanced microvascular hemostasis with low systemic absorption and less systemic side effects.

However, the dosage and concentration of TXA solution used in topical application show no clear-cut guidelines in studies. Existing data on clinical efficacy and safety of topical TXA administration compared with placebo in TKA have been confirmed in many RCTs with various dosages (1.5 to 3 g) and topical routes of administration (Table 4). Some studies demonstrated that IA delivery of > 2-g TXA can effectively reduce blood loss and transfusion requirements [44]. Moreover, a recent meta-analysis has confirmed that topical administration of > 2-g TXA is a safe and simple alternative for patients with high risk of thromboembolic complications [45]. At our institution, a well-conducted RCT by Yue et al. has demonstrated that a high dose (3 g) of topical TXA appears to represent an effective and safe way to stop bleeding and transfusions [31]. Also, Huang et al. has demonstrated that a regimen with combined topical high-concentration TXA solution (1.5 g TXA diluted in 50 mL normal saline) and 1.5 g of IV TXA was effective in reducing the blood loss. This trial is a continuation of these previous studies, and compares efficacy of topical dose of 3 g of TXA with multiple doses of oral TXA administration.

**Table 4:** Overview of relevant randomized controlled trial regarding Oral and IA administration of TXA compared with placebo in total knee arthroplasty

Authors	Year	Dosing regimens	No. of patients		Reduced blood loss	Reduction of Hb	Increased complications	Reduced transfusion
			TXA	Control				
Oral administration								
Zohar et al.	2004	1 g oral TXA before surgery, 6 h, 12 h, and 18 h	20	20	Significant	NA	NS	Significant
Charoencholvanich et al.	2011	10 mg/kg before deflation; 0.5 g oral TXA for 5 days	50	50	Significant	Significant	NS	Significant
Alipour et al.	2013	1 g oral TXA before surgery, 6 h, 12 h, and 18 h	26	27	Significant	NA	NS	NA
Lee et al.	2017	1 g oral TXA 2 h before surgery, 6 and 12 h	95	95	Significant	Significant	NS	NA
Yuan et al.	2017	20 mg/kg oral TXA 2 h before surgery; 2 g oral TXA 12 h postoperatively	140	140	NA	Significant	NS	NA
IA administration								
Wong et al. (1)	2010	1.5 g/100 mL after cement	33	35	Significant	Significant	NS	NS
Wong et al. (2)	2010	3 g/100 mL after cement	31	35	Significant	Significant	NS	NS
Ishida et al.	2011	2 g/20 mL at closure	50	50	NA	NA	NS	NS
Sa-Ngasoongsong et al.	2011	0.25 g/25 mL at closure	24	24	Significant	Significant	NS	Significant
Onodera et al.	2012	1 g/50 mL after closure	50	50	Significant	Significant	NS	NS
Roy et al.	2012	0.5 g/5 mL	25	25	NA	Significant	NS	NS
Alshryda et al.	2013	1 g/50 mL at closure	79	78	Significant	Significant	NS	Significant
Georgiadis et al.	2013	2 g/75 mL	50	51	Significant	Significant	NS	NS
Sa-Ngasoongsong et al. (1)	2013	0.25 g/25 mL after closure	45	45	Significant	Significant	NS	NS
Sa-Ngasoongsong et al. (2)	2013	0.5 g/25 mL after closure	45	45	Significant	Significant	NS	NS
Martin et al.	2014	2 g/100 mL prior to closure	25	25	NA	Significant	NS	NA
Lin et al.	2015	1 g/20 mL prior to closure	40	40	Significant	Significant	NS	Significant
Wang et al.	2015	0.5 g/10 mL prior to closure	30	30	Significant	Significant	NS	Significant
Yang et al.	2015	0.5 g/20 mL prior to closure	40	40	Significant	Significant	NS	Significant
Maniar et al.	2012	3.0 g/100 mL before deflation for 5 mins	40	40	Significant	NA	NS	NS
Seo et al.	2013	1.5 g/100 mL	50	50	Significant	NS	NS	Significant
Sarzaeem et al. (1)	2014	3.0 g TXA/100 mL before suturing	50	50	NA	NS	NS	NS
Sarzaeem et al. (2)	2014	1.5 g TXA/100 mL after closure	50	50	NA	Significant	NS	NS
Aguilera et al.	2015	1.0 g/10 mL after cement	50	50	Significant	NS	NS	NS
Cavusoglu et al.	2015	2.0 g/100 mL before closure	20	20	NA	NA	NS	NA
Digas et al.	2015	2.0 g TXA after closure	30	30	Significant	NS	NS	NS
Oztas et al.	2015	2.0 g TXA	30	30	Significant	NA	NS	Significant
Drosos et al.	2016	1.0 g/30 mL	30	30	Significant	NS	NS	Significant
Keyhani et al.	2016	1.5 g/50 mL before closure; 1.5 g/50 mL after closure	40	40	Significant	Significant	NS	NS
Tzatzairis et al.	2016	1.0 g/100 mL after closure	40	40	Significant	Significant	NS	Significant
Prakash et al. (1)	2017	3 g/50 ml prior to closure	50	50	NA	NA	NS	NS
Prakash et al. (2)	2017	3 g/50 ml after closure	50	50	NA	NA	NS	NS
Song et al.	2017	3 g/50 ml after closure	50	50	Significant	Significant	NS	NS
Stowers et al.	2017	1.5 g TXA	59	21	Significant	NA	NS	NS
Ugurlu et al.	2017	1.5 g/50 mL before closure; 1.5 g/50 mL after closure	42	41	NA	Significant	NS	NS
Yuan et al.	2017	3 g/60 ml after closure	140	140	NA	Significant	NS	Significant

*NA* not available, *NS* not significant

Data on comparison of the effectiveness of oral TXA administration and placebo on blood loss, reduction of Hb, and transfusions are summarized in Table 4. Although oral application of TXA was effective in reducing blood loss, as also confirmed in a recent meta-analysis [46], to our knowledge, limited research has been conducted on the route of the less expensive oral form of TXA administration in TKA. A 4-armed RCT by Zohar included four treatment groups (i.e., IV TXA, oral TXA, IV plus oral TXA, and placebo) and demonstrated that oral application of TXA (1 g before surgery and repeat every 6 h for the next 18 h) was superior to IV TXA administration due to significant difference in blood savings and ease of oral drug administration [15]. Fillingham et al. performed a RCT containing only 71 patients with the treatment groups of IV TXA and oral TXA. They found that a single gram of oral TXA 2 h before incision can provide an equivalent reduction in Hb level and blood loss in comparison with IV medicine administration [47]. In spite of the blood-sparing efficacy and potential cost-saving benefits of oral TXA, some concerns do remain about thromboembolic complications after oral administration in high-risk patient population. In addition, a tourniquet and postoperative drain were applied as a standard practice in the abovementioned studies. Thus, it remains unclear whether the efficacy and safety of oral TXA is equal to or less than that of IA TXA in TKA without a tourniquet and drain.

Some drainage-related factors, such as the gradually declining hematocrit in drain output and residual blood in the drain, could have an effect on the accuracy of blood loss measurement [48]. A RCT conducted by Wang et al. [36] demonstrated that the postoperative drainage provided no clear benefits in blood loss, knee function, and early recovery in primary TKA. Moreover, Huang et al. performed a RCT of application of tourniquet in TKA at our institution and reported that tourniquet application may cause muscle damage and delay strength recovery [22]. Also, a recent meta-analysis has confirmed that tourniquet could increase the risk of thromboembolic events [49]. Some RCTs have demonstrated that the lack of tourniquet did not affect the tibial cement mantle thickness [50] and short-term fixation [51]. Thus, the tourniquet and postoperative drain were not applied after surgery routinely. The tourniquet-related fibrinolysis may have a substantial effect on bleeding kinetics, which is related to the assessment of the effect of oral TXA administration when a tourniquet is not utilized in the procedure.

Although our trial had a similar goal of comparing oral and other routes of TXA administration as the above-mentioned studies, we assessed the efficacy of perioperative standardized oral TXA versus IA TXA alone for unilateral TKA, conducted in a fast-track setup, without the use of a tourniquet and postoperative drains. The dosing and timing of oral regimes were based on pharmacokinetic and serum studies, and therapeutic levels can be maintained for approximately 12 h after surgery, which covers the most period of postoperative hyperfibrinolysis. The major finding of our study was that oral TXA administration provided an equivalent blood-saving benefit when compared with IA administration of 3 g of TXA.

Blood-conservation strategy during “enhanced recovery” knee replacement, as part of multimodal protocol, has substantially affected costs by decreasing the incidence of morbidity and mortality, transfusion-associated complications, and hospital stay. The finding was supported by a retrospective economic analysis of TXA application, which indicated $879 direct savings per joint surgery with use of TXA [52]. Moreover, a retrospective analysis by Irwin et al. [29] contained 2698 patients undergoing total joint arthroplasty and demonstrated that the patients receiving the substituted 2-g oral TXA bolus had a lower risk of blood transfusion, with great cumulative savings (£29,788), when in comparison with 15-mg/kg IV administration. Retrospective clinical and cost-benefit evaluation of IA TXA administration have shown an estimated cost savings of $1500 per patient with decreased transfusion costs [53]. A RCT by Kayupov et al. reported that an appropriate oral dose can save $33 to $94 compared with an equivalent dose depending on the TXA formulation and administration route selected [20]. In China, more than 200,000 primary TKAs were conducted annually. Considering aging population and longer life expectancy, the number of TKAs will increase dramatically over time [54]. Thus, a transition to oral TXA may lead to great cost savings per year for health-care system.

No significant differences identified in transfusion rates and thromboembolic complications (i.e., DVT and PE) in this current study. However, there is substantial evidence supporting the safety of oral and IA application of TXA with no additional thromboembolic complications. In our study, there was no significant differences between the two groups regarding knee function and quality of life, which were tested with use of range of motion, knee society score, pain score, and QoR-40 measures after surgery.

## Conclusions

This prospective randomized controlled trial in the setting of total knee arthroplasty using a fast-track protocol, with no tourniquet and postoperative drain, demonstrated that oral administration of TXA (2-g TXA before incision and 1 g TXA every 6 h for 12 h after surgery) provided an equivalent blood-saving benefit compared with 3 g of IA TXA administration with great cost savings and no increased thromboembolic events.

## Abbreviations

IA: Intra-articular
IV: Intravenous
RCT: Randomized controlled trials
TKA: Total knee arthroplasty
TXA: Tranexamic acid
